# Supplementary material for: A triangular model of fractal growth with application to adsorptive spin-coating of polymers
Source: PLoS One. 2024 Feb 23;19(2):e0298916. doi: 10.1371/journal.pone.0298916 (PMC10889878; doi:10.1371/journal.pone.0298916)
Supplement: Attachment — Submitted filename: Response to Reviewers.docx [file pone.0298916.s004.docx]

**Modifications to meet journal requirements:**

1. Headings, figure references, figure captions, and the title page have all been modified to meet PLOS ONE’s style requirements.
2. We have removed a declaration of funding from our Acknowledgements section. We do not have any modifications to make to our original Funding Statement.
3. Minimal underlying data have been added as supplementary information. In particular, the data behind Figures 5, 8 and 10 have been added as separate spreadsheets.
4. An updated copyright permission form has been added to our materials. This is what the American Chemical Society provides when there is no charge for the reproduction of copyrighted material. I confirmed this by talking with the organization that handles their copyrights. I would also note that one of the authors of that work is a coauthor on this article (WC) and she was responsible for producing the original images. We have modified the figure captions as requested.

**Modifications and responses to specific critiques by the reviewers:**

Reviewer 1

**Reviewer:** In the introduction, the wording is confusing: What are the normal rules of geometry? Do the authors mean Euclidean Geometry (line 44)? Which objects do they refer to generally having dimensions 1,2, or 3 (lines 44-45)? Objects in the physical world? Hausdorff dimension can be generalised to Rn, so it is important to clarify this point.

**Response:** We have modified this section to clarify that we are referring to Euclidean geometry and idealized geometrical objects (lines 43 – 44).

**Reviewer:** Which type of fractal dimension is discussed here? Capacity? Correlation? Information? I guess it’s capacity (box-counting), but please confirm.

**Response:** We have clarified in the introduction that we are referring to Minkowski-Bouligand dimension (line 45).

**Reviewer:** The claim that this model is comparable in simplicity to the DLA does not hold. In its original definition, the DLA starts with a seed, and a particle undergoes a random walk until it “touches” the seed. The process repeats with one particle walking at each iteration. That’s it, no more parameters. The presented model contains several parameters, which create a non-trivial phase space to analyse.

**Response:** The reviewer is correct in noting that our model is not as simple as the DLA model. However, we believe it is still conceptually quite simple. We also do not believe that the reviewer’s characterization of the DLA model’s simplicity is quite accurate. The nature of the random walk in terms of turn angle and step size both impact the resulting structure. We have added qualifying and clarifying language to the places where we compare the complexity of our model to the DLA model (lines 30, 95 – 97, 476 – 477, 531 – 532 and 577).

**Reviewer:** There are some suggestions in this work of what to expect if some parameters change (section III.A) and Figure 5, but nothing systematic. The authors must study the phase space to understand this model and its full potential. I am not talking about the full combinations of all the elements of Table 1, but at least Probability of bonding and Maximum bonding distance, in my view, are the critical parameters. Mobility period also seems quite important, but it is unclear from the text how it works (more about this later). Authors need to perform several runs for many different combinations of these parameters. For example, run 100 experiments with bond-rate = i and bond-range = j, where i in [0.01,0.02,…,0.1] and j in [0.1,0.2,0.3,…,1], i.e., 100 for bond-rate=0.01 and bond-range=0.1; 100experiments for bond-rate=0.01 and bond-range=0.2, and so on. At the end, you’ll have 10,000 experiments to study this limited phase space. Only then can we start to understand the model. Each experiment would produce the four statistics from section III.B statistics, and then the authors need to get the mean value for each combination.

**Response:** The reviewer is correct in their characterization of the correct way to explore the behavior of a model. We did conduct a systematic review of phase space, the results of which were reported in the previous Figure 5. Both bond-range and mobility varied over a range of variables, and 6 replications were done for each combination of values. Figure 5 reported the means of each set of trials for each statistic. We repeated this for three different settings of bond-rate.

Our search points were not as dense in phase space as the reviewer suggests, nor did we have the focus on bond-rate that the reviewer suggests would be important. Instead, we incorporated a gradient for mobility. Our choices were informed by significant exploratory work prior to our systematic runs that guided our selection of parameter settings and gave us confidence that we explored a range of phase space that covered all observable model behaviors.

That said, we have repeated our previous work, this time with 50 replications, and have changed Figure 5 to show updated values. The reported means did not change significantly, and more importantly, the observed trends were consistent with our previous findings.

**Reviewer:** The model definition is acceptable, except for the contraction step. I understand the general idea behind the parameter, but the mobility concept is unclear. How are the linked neighbours defined? This line, “a displacement equal to 0.01 times each of the vectors represented by directed links from the particle to each of their linked neighbors,” makes little sense. What is 0.01 time in this context? Maybe it is only how it is redacted, but it needs to be explained better.

**Response:** We acknowledge the ambiguity in this section, and we have revised it for greater clarity (lines 144 – 165). In particular, we defined a vector equation for determining the displacement of each particle during the contraction step.

**Reviewer:** In the introduction, there is a strong emphasis on the fractal nature of these models. However, this characteristic dilutes as the work advances, only to be mentioned again in section III.B, and there is no mention of why it’s relevant for this type of aggregates.

**Response:** Models of fractal growth have been of interest for many decades and remain so. Vicsek’s Fractal Growth Phenomena (Vicsek 1992) continues to be heavily cited 30 years after publication. We present this model as a fractal growth model and believe it fits within that general body of research. Given the demonstrated fractal dimension of the model output, we did not feel further justification was necessary. The perceived dilution in our discussion of the fractal nature of our model is due to the fact that we spent a significant amount of time defining and demonstrating the model as well as determining other characteristics of the model besides its fractal characteristics. Fractal dimension alone is not enough to describe the range of behaviors of the model nor to calibrate the model to different empirical settings, but it is an important statistic for both objectives.

**Reviewer:** Besides the lack of a rigorous statistical treatment of the model, the other major problem of this piece is how the fractal dimension is calculated. Working over the images creates a serious bias in the values. The reported dimension has a strong correlation with the image resolution. As the authors generate the model, they have all the exact positions for each particle, so they need to use the box-counting using these positions. There are plenty of codes for doing that; no need to create an image and pass it through ImageJ. In a revised version, I suggest sharing such code or a pseudo-code as part of the manuscript.

**Response:** This is an issue that merits continued consideration. However, the reviewer’s proposed solution is not appropriate to our model since we do not model points (particles) but rather triangles composed of line segments (bonds). Theoretically, there are ways to directly code a box-counting algorithm using the endpoints of the segments, but we do not yet have a solution to this issue. However, whether or not there is a need for this depends upon the usage of the model. Analyzing the model as a mathematical structure makes such an algorithm desirable. But if the model has value in terms of simulating empirical phenomena, then it needs to be able to simulate the available representation of those phenomena which in the case of spin-coated polymers (and many others) will be in the form of images.

We have made a note in the text (lines 312 - 317) regarding the issue raised and the steps we have taken to make sure that dimension is consistently applied across images by standardizing plotting parameters including image scale and resolution.

**Reviewer:** The text mentions the concept of Lacunarity, which is never defined or cited. I fail to see the value of the Lacunarity measures in this work in its present form. I suggest removing it completely or properly defining and better explaining its importance for the model.

**Response:** We have added a description and a citation for Lacunarity and explain that we use it to demonstrate the correspondence between our model output and spin-coated fractals following the methodology used in that work (lines 383 - 389). We decided not to provide a rigorous definition as it would add little to demonstrating the correspondence between our model and polymer fractals and would be complex and uninteresting to most readers.

**Reviewer:** Better images are needed. Figure 1 is good, except, again, at the contraction frames. I suggest showing a zoom section for Figures 2 and 3 to visualise the actual triangles and the local structures they form.

**Response:** Figures 2 and 3 have been modified according to the reviewer’s suggestion. We added text to Figure 1 to clarify what is being shown in the contraction steps (lines 162 – 165).

**Reviewer:** At line 500, it is mentioned that variable dimension often comes at the cost of increased complexity. What does this mean? That Higher dimension  Higher complexity? Or vice versa. How is complexity defined? These are not trivial questions. I suggest removing all lines from 500 to 509, as those add nothing but confusion to this work.

**Response:** We believe that it is an important point to make that our model is able to produce variable fractal dimensions while remaining conceptually simpler than comparable models. We acknowledge that assessing model complexity rigorously is not a trivial task and that our arguments are more qualitative than quantitative. We have added clarifying text (lines 521 – 529) to address some of the reviewer’s questions, and we believe that further consideration of the matter may be left to the reader.

Reviewer 2

**Reviewer:** “1) It would be interesting to have a better look at the early stages of the pattern formation, shown in Figures 2 and 3. What are the authors representing in Figures 2 and 3? The particles or the bonds?”

**Response:** We have added additional discussion of Figures 2 and 3 (lines 172 - 181) including clarification of what exactly is being plotted.

**Reviewer:** “2) Would it be possible to compute quantities like a radial distribution function, which could provide additional information about the fractal structure of the patterns?”

**Response:** We define several statistics that we believe provide an effective description of model variability. However, following the reviewer’s suggestion, we computed radial distribution functions for several different combinations of model parameters based on pixelated model output. Model output did not show any radial variation in density which is consistent with the model definition which begins with a distribution of particles that has uniform radial density.

**Reviewer:** “3) Did the authors try to extend the work to 3 dimensional systems, using tetrahedra instead of triangles?”

**Response:** We have made some initial (and successful) forays into a 3D version of the model, and we describe this at the end of the discussion section (lines 568 - 571). Given the preliminary nature of this work, we do not provide additional details at this time.
